# Public Funding and Open Access to Research: A Review of Canadian Multiple Sclerosis Research

**DOI:** 10.2196/jmir.6250

**Published:** 2017-02-27

**Authors:** Caitlin Bakker, Carol Stephenson, Erin Stephenson, Debbie Chaves

**Affiliations:** ^1^ Health Sciences Libraries University of Minnesota Minneapolis, MN United States; ^2^ Council of Prairie and Pacific University Libraries Vancouver, BC Canada; ^3^ Hotchkiss Brain Institute and the Department of Clinical Neurosciences University of Calgary Calgary, AB Canada; ^4^ Library Wilfrid Laurier University Waterloo, ON Canada

**Keywords:** multiple sclerosis, open access publishing, research support as topic

## Abstract

**Background:**

Multiple sclerosis (MS), a progressive demyelinating disease of the brain and spinal cord, is the leading cause of nontraumatic neurological damage in young adults. Canada has one of the highest reported incidents of MS, with estimates between 55 and 240 per 100,000 individuals. Between 2009 and 2014, the MS Society of Canada provided over Can $90 million to researchers and, since 2013, has encouraged researchers to make both current and previous research products openly available.

**Objective:**

The goal of the study was to determine the open access (OA) cost implications and repository policies of journals frequently used by a sample of MS researchers. This study benchmarked current publishing preferences by MS Society of Canada researchers by examining the OA full-text availability of journal articles written by researchers funded between 2009 and 2014.

**Methods:**

Researchers were identified from the 2009 to 2014 annual MS Society of Canada Research Summaries. Articles were identified through searches in Web of Science, Scopus, Medline and Embase (both via OVID). Journal level analysis included comparison of OA policies, including article processing charges (APCs) and repository policies. Data were analyzed using descriptive statistics.

**Results:**

There were 758 articles analyzed in this study, of which 288 (38.0%) were OA articles. The majority of authors were still relying on journal policies for deposit in PubMed Central or availability on publisher websites for OA. Gold OA journals accounted for 10.2% of the journals in this study and were associated with significantly lower APCs (US $1900) than in hybrid journals (US $3000). Review of the journal self-archiving options highlighted the complexity of stipulations that authors would have to navigate to legally deposit a version of their article.

**Conclusions:**

This study found that there are currently researcher- and publisher-imposed barriers to both the gold and green roads to OA. These results provide a current benchmark against which efforts to enhance openness can be measured and can serve as a reference point in future assessments of the impact of OA policies within this field. With funding agencies worldwide releasing OA mandates, future success in compliance will require changes to how researchers and publishers approach production and dissemination of research.

## Introduction

Multiple sclerosis (MS), a progressive demyelinating disease of the brain and spinal cord, is the leading cause of nontraumatic neurological damage in young adults [1]. Depending on the affected areas of the brain, balance, vision, hearing, thinking, and memory may ultimately be impacted. Canada has one of the highest reported incidents of MS, with estimates between 55 and 240 per 100,000 individuals [2]. Despite over 200 years of research and significant recent findings [3], a standard, evidence-based treatment proven to halt the chronic progression and long-term disability has remained elusive. Given the debilitating character of MS and its prevalence in Canada, it is not surprising that the MS Society of Canada infuse significant funding to advance research. Between 2009 and 2014, the MS Society of Canada provided over Can $90 million to researchers in four major research areas: (1) symptom management and quality of life, (2) progression and therapies, (3) cause and risk factors, and (4) nerve damage and repair [4].

The MS Society of Canada has strongly encouraged open access (OA) and the broad dissemination of research. The MS Society of Canada’s OA policy, which came into effect in July 2013, requires grant recipients to make every effort to ensure their peer-reviewed publications are available OA within 6 months of publication [5]. While the policy came into effect in 2013, the MS Society of Canada encourages retroactive compliance for research funded before that date.

The information needs and information-seeking behaviors of clinicians and patients with MS have been well documented [6-10]. Studies have shown that MS patients are demanding active roles in their treatments and the decision-making process [11-13], and health care information on the Internet ranks second to health professionals as a source of information for patients [14]. Although scientists and funding agencies recognize the potential public value of research and of making work available OA, resources are required to facilitate the process. Whether these are financial resources to pay for article processing charges (APCs) or the time to negotiate with journals to allow for deposit in an appropriate repository, OA requires work on the part of the researcher. The appropriate allocation of resources for broad dissemination remains a question.

Using the MS Society of Canada as a case study, this paper examines the OA full-text availability of scientific articles by Canadian researchers funded between 2009 and 2014, including the publishing venues and associated costs of making work openly available. The goal of the study was to determine the extent of OA, the cost implications, and repository policies of journals frequently used by MS researchers as an assessment of the current context which can be used in future evaluations of the extent of openness and the effectiveness of mandates in making research OA.

## Methods

### Database Search

Researchers were identified from the 2009 to 2014 annual MS Society of Canada Research Summaries [15]. Researcher names and affiliations, titles of the research projects, funded amounts, and funded years were recorded in an Excel (Microsoft) file. The name of the researcher and the keyword “Multiple Sclerosis” were searched in 4 databases: Web of Science, Scopus, and Embase and Medline (both via OVID platform). Due to the inconsistencies in funding information in journal metadata, searches did not incorporate the MS Society of Canada as a funding body. In selected cases, an author affiliation was used to aid in disambiguation. Publications were limited to the first year the researcher received MS Society funding and all subsequent years. Articles published in 2015 were included in this analysis to account for the time necessary to finalize research, write publications, and complete the publishing process. Publication types were limited to journal articles, review articles, and conference papers reproduced in their entirety in a journal, including items that were both published and in press. Searches were limited to English language only.

The results from the database searches were merged into a single, deduplicated file of articles. A secondary quality check was performed by manually reviewing the article details to ensure relevance and accuracy. The article title was searched in Google and PubMed to determine if the full-text was openly accessible (open access) either through the publisher website or PubMed Central. The journal policy for access through PubMed Central and the version of the article on PubMed Central (author manuscript to meet compliance with a funder policy or a journal OA policy) were recorded. Full-text access through other sources such as Academia.edu or ResearchGate was not included because these sites do not necessarily guarantee that the full-text option is a legitimate copy.

### Colors to Categorize Policies

Subscription policies were used to identify the title as a pure gold journal or a hybrid journal. The self-archiving policies for each journal were obtained from SHERPA RoMEO, a UK academic supported database that provides information regarding copyright policies and rights retained by the authors when publishing in specific academic journals. SHERPA RoMEO uses colors to categorize publishers’ archiving policies: white (archiving is not formally supported), yellow (authors can archive preprint, that is, prerefereeing), blue (authors can archive postprint, that is, final draft postrefereeing, or publisher's version), or green (authors can archive preprint and postprint, or publisher's version). Each journal in the study was assigned one of the 4 colors. The OA policies were analyzed first by publisher, per journal, and then at the article level.

For each journal, APC policies were searched on the publisher website. APC costs were also converted to US dollars. The maximum APC fee quoted by the journal was recorded where there were variable fees listed. APCs fees were variable depending on the type of article, memberships, institutional affiliations, funding bodies, or which Creative Commons license was selected.

## Results

### Overview of Publishing Venues

The MS Society of Canada funded 77 Canadian researchers between 2009 and 2014 in one of their four major research areas. This study identified a total of 758 MS related articles in 211 journals produced by 61 publishers. Of the 758 articles, 288 (38.0%) were OA articles produced in 93 journals by 42 publishers.

**Figure 1:**
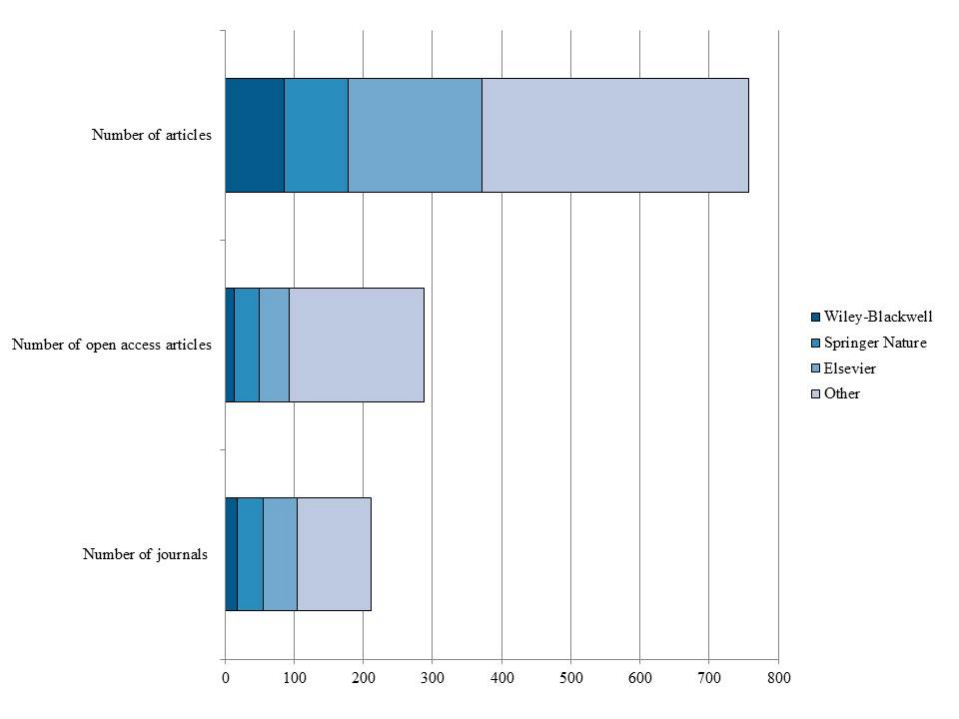
The top three publishers by journal and article count.

When article distribution per journal was analyzed to identify the preferred or most popular publishing venues, there was a noticeable difference in the dominance of publishers. Three major publishers (Elsevier, Wiley, and Springer Nature) dominated the research output, with 105 of 211 journals (49.7%) and 374 of 758 articles (49.3%). However, these publishers only accounted for 93 of the 288 OA articles (32.2%) in the study (Figure 1). Only one Elsevier journal (*NeuroImage: Clinical*) and 3 Springer journals (*BMC Health Services Research*, *BMC Neurology*, and *Journal of Neuroinflammation*) were pure gold in which every article was OA full-text upon publication. There were no pure gold journals from Wiley-Blackwell identified in the articles analyzed in this study.

A total of 19 journals from 13 publishers accounted for 388 of the 758 articles (51.2%) and 146 of the 288 OA articles (50.7%) in the study (Table 1). The three major publishers (Elsevier, Wiley, and Springer Nature) published 8 of the 19 journals in this preferred grouping, but SAGE and Lippincott Williams and Wilkins (LWW) were the top publishers with both their journals, *Multiple Sclerosis* (SAGE) and *Neurology* (LWW) accounting for 142 of the 758 articles (18.7%) in the study. Only one pure gold journal (*PLoS ONE*) was in this grouping, ranking 11th, with 14 of the 758 articles in the study (Table 1).

**Table 1:** The most frequently used journals, accounting for 51.2% of the articles. Number of articles, number of open access (OA) articles, article processing charges (APC), and SHERPA RoMEO color are provided.

Journal title	Articles (n)	OA articles (n)	Maximum APC (US $)	SHERPA RoMEO color
*Multiple Sclerosis*	76	15	3000	Green
*Neurology*	66	35	3100	Yellow
*Journal of the Neurological Sciences*	23	0	2500	Green
*Annals of Neurology*	23	4	3000	Yellow
*GLIA*	19	2	3000	Yellow
*Journal of Neuroimmunology*	18	2	2500	Green
*Multiple Sclerosis and Related Disorders*	18	2	2500	Green
*Journal of Neurology*	17	7	3000	Green
*Journal of Immunology*	16	14	3000	Blue
*Lancet Neurology*	16	0	5000	Green
*PLoS ONE*	14	14	1350	Green
*Canadian Journal of Neurological Sciences*	13	0	2000	Green
*NeuroImage*	13	2	2200	Green
*Journal of Neurology, Neurosurgery and Psychiatry*	10	7	2822	Green
*Journal of Neuroscience*	10	10	2820	Yellow
*International Journal of MS Care*	9	9	n/a	Ungraded
*Neuro-epidemiology*	9	6	3250	Green
*Brain*	9	7	3200	Yellow
*JAMA Neurology (Archives of Neurology)*	9	8	n/a	White

### Article Processing Charges

**Figure 2:**
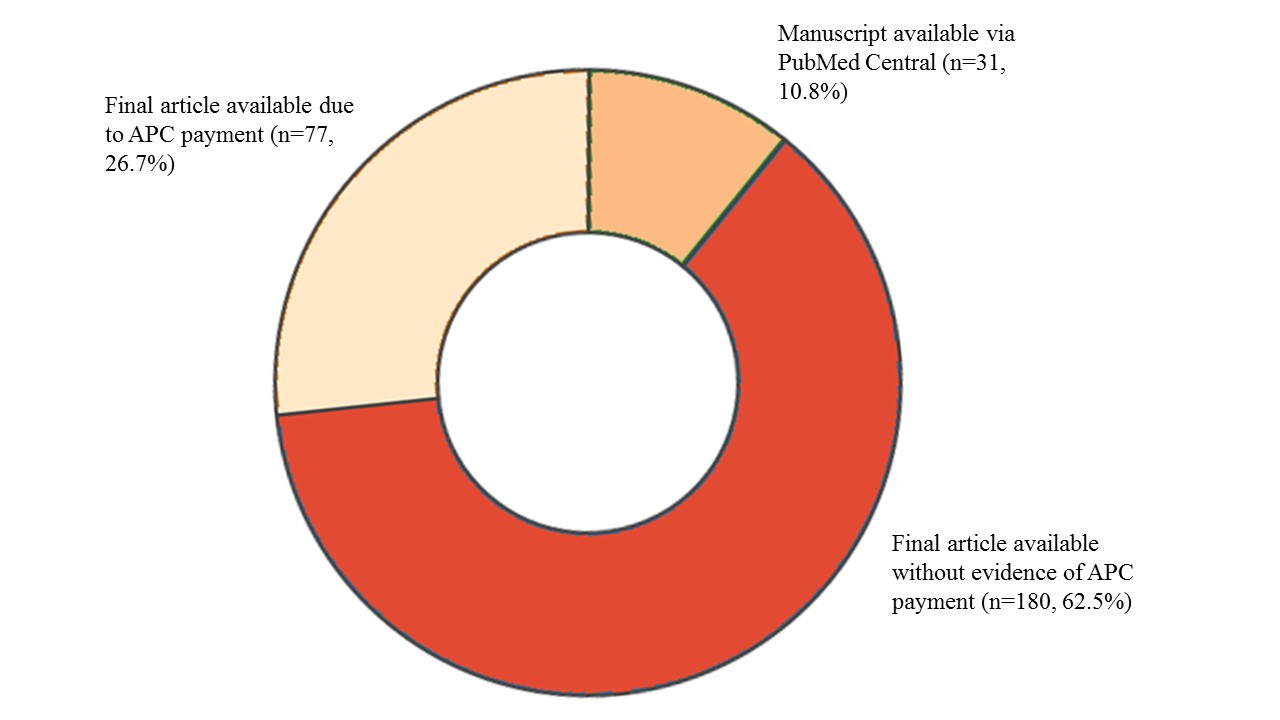
The number of final articles available as open access due to article processing charge (APC) payments, article manuscript deposit in PubMed Central, and final articles available without evidence of APC payment through deposit of published articles to PubMed Central or availability from the publisher website.

A closer examination of the reason for the availability of OA revealed limited use of APC payments or deposit of author manuscripts in PubMed Central, even though most journals offered these as options (see Figure 2). Of the 288 OA articles, 180 (62.5%) were made available in their final typeset form, either through deposit in PubMed Central (163/180, 90.6%) or through the publisher’s website (17/180, 9.4%) without evidence of APC payment. In comparison, author directed OA through APCs accounted for 77 of the 288 articles (26.7%), whereas 31 of the 288 articles (10.8%) were made available as manuscripts deposited in PubMed Central. The limited use of APCs is even more striking when all articles in the study are considered. Whereas 662 of the 758 articles were published in journals with APC options, only 77 of the 758 (10.2%) have been published using that option.

APCs for pure gold and hybrid journals revealed significant differences in potential costs to authors. Of the 211 journals in this study, 22 were pure gold, with each of the 60 articles produced by these journals freely available full-text upon publication. Pure gold journals not only use APCs to cover publication costs but also use institutional memberships or other funding options to provide all content openly accessible upon publications. The pure gold journals in this study had variable APCs depending on memberships, government support, and the type of article published. APCs were listed on publisher sites for 19 of the 22 journals. One of these unlisted journals (*Preventing Chronic Disease*) was produced by the Centers for Disease Control and Prevention which does not use APCs. *Functional Neurology* and *European Neurological Review* also did not list APCs in their author instructions. For the 19 journals for which APCs were found, the average and mode fees were US $1900.

APCs were found for 137 of the 189 hybrid journals. The other 52 journals either required authors to contact the publisher for fee information or it was not evident if the option existed. Fees within a journal could vary depending on memberships, institutional affiliations, funding bodies, or which Creative Commons license was selected. The APCs ranged from US $600 to US $5000. The APC fees were notably higher for hybrid journals than for pure gold journals. The average cost was US $2800, and the mode was US $3000. The APCs for the hybrid journals with the highest number of articles in this study ranged from US $1000 for members in the *Canadian Journal of Neurological Sciences* to US $5000 for *Lancet Neurology*. Table 2 provides a profile of the potential APC costs for articles from this study published in 2014.

**Table 2:** Snapshot of maximum article processing charges (APCs) for articles published in 2014.

Journal Type	N		APC^a^ Information		
	Journals	Articles	Range	Average	Mode
Journals without APC information	18	22	N/A	N/A	N/A
Gold journals with APCs	9	16	US $1350 to US $2310	US $1735	US $1500
Hybrid journals with APCs	54	108	US $600 to US $5000	US $2979	US $3000

^a^APC: article processing charge.

### Policies for and Prevalence of Self-Archiving

With the impracticability of the APC model for many researchers, the policies for self-deposit were reviewed for the 211 journals in the study. The SHERPA RoMEO website provided information on journal policies for 203 of 211 journals in the study. The ability for authors to self-archive a preprint or postprint of the article to an institutional or subject-based repository was offered by the majority of journals, but there was large variability in the version of the manuscript that could be deposited.

Of the 211 journals, 120 allowed deposit of the final draft (postprint) of the manuscript, 71 allowed the final draft but with additional restrictions, and 11 did not allow the final draft and information was not provided the other 9 journals. The SHERPA RoMEO analysis identified deposit embargoes on 138 of the journals that would have affected 564 of the 758 articles in this study (74%). In addition, the restrictive self-archiving policies (yellow and white SHERPA RoMEO categories) were applied to journals produced by 16 societies.

Of the 77 MS Society of Canada funded researchers included in this study, 62 had affiliations that would offer access to an institutional repository (IR). A secondary search of repositories for the 760 articles found that 8 of the 760 articles (1%) identified in this study were made available through an IR. Of these, all 8 were originally published in pure gold journals, namely in BioMed Central titles. IRs did not appear to function as a mechanism to make otherwise inaccessible content publicly available.

## Discussion

### Principal Findings

Despite encouragement from the public and the clinical community [16-18], and the MS Society of Canada’s goal of making both current and previous research publicly available, the overall rates of openness in MS research remains low. Of the 758 articles included in this study, only 288 are currently available OA. This finding acts as a current benchmark against which the efforts to enhance openness and dissemination can be measured, and can serve as a reference point when assessing the impact of OA policies in this field.

### The Cost of Open Access

The hybrid gold model is fraught with cost implications to both the individual researcher and institutions. The monopoly of hybrid journal ownership by the big three commercial publishers (Elsevier, Wiley, and Springer Nature) has serious implications for the openness of research findings. In this study, these 3 publishers controlled 49.7% of the journals (105/211), 49.3% of the article output (374/758), and the majority (96/105, 91.4%) of their journals followed the hybrid model.

The hybrid model had been developed as a compromise between subscription publishers and OA advocates [19]. However, it has underperformed, with less than 2% of eligible authors making work available in this manner [20]. Our study confirmed the low use of APCs, with 10% of eligible articles being made available with this option. In a recent survey of Canadian scientists, 78% felt that publishing OA was unaffordable and 86% felt that funding for OA publishing was not readily available [21]. Recent studies continue to report that lack of funds for APCs is an impediment to publishing in OA or hybrid journals [22-24].

The mode APC cost for hybrid journals was US $3000, which was 45% higher than for pure gold journals, a finding which is consistent with other recent research [25,26]. The impact of high APC costs and institutions costs has notable economic consequences in Canada, where the majority of researchers and universities are publicly funded. Academic libraries pay publishers large fees for institutional subscriptions to journals then authors pay the same publishers additional APC costs to publish their article in those same journals. In effect, there is “double dipping” of public funding, especially in the context where Canadian universities are subscribing to the “big journal deals” with all the major publishers, through the Canadian Knowledge Research Network (CRKN). In this Canadian-context study, it is also important to consider that these figures represent US dollar calculations and that exchange rate fluctuations add additional burdens to fund APCs.

### Pure Gold Publication Patterns

The pure gold road to OA is not a road well-travelled by MS researchers. Pure gold journals accounted for 7.9% (60/758) of the articles and 10.4% (22/211) of the journals in this study. Although this study showed evidence of the use of pure gold journals by the researcher, the low number of articles in these venues may indicate that MS researchers feel that OA is poorly regulated, of poor quality, and lacking in peer review. This would support previous research that has found that despite awareness of OA and OA issues, faculty concerns about quality, reputation, copyright, plagiarism, and a perceived lack of peer review remained constant [27,28]. The appearance of *PLoS ONE*, ranked 11th based on article count of the 211 journals, is a promising evidence of the growing influence of gold OA.

### Barriers to Green Open Access

Self-archiving is the most cost-effective method of providing OA to research findings for researchers. Local self-archiving includes depositing a postprint or preprint of the article in an IR or self-archiving in a subject repository. However, rather than offering a simplified option, publishers have created barriers to OA through unsustainable complexities involved in self-archiving policies. Of the 35 professional society or association journals represented in this study, 15 had restrictive yellow self-archiving policies. Yellow self-archiving only allows authors to post the prerefereed preprint. With peer review considered the most important criteria in journal publishing, there is potentially little value to the open communication of research of an article without peer review [29,30].

Poltronieri et al [31] also found that in their survey of journals, more than half of the publishers are still imposing yellow and white restrictions on self-archiving. Even among green journals, navigating restrictions and permissions is challenging. In addition to the version requirements of the publisher, there were additional requirements regarding embargo periods, restricting when the self-archived version could be made available. The journal embargoes were often listed as a set of complex conditions that authors would need to navigate to comply with the journal’s requirements for self-archiving.

### Future Directions

The solution to resistance, either from journals or researchers, is sometimes assumed to be the requirements from funding sources or institutions [32,33]. Mandated public access from funding agencies, principally National Institutes of Health (NIH), has led to tremendous growth in the availability of biomedical literature [34]. However, the NIH, despite having an OA policy written into law 2008, found that low compliance continued to be a major issue. Researchers had indicated that a lack of time, a frustrating deposit process, and confusing journal policies were the primary reasons for lack of compliance [35]. NIH introduced a policy delaying applications and funding if publications associated with the research were not in compliance. Following this, NIH saw aggregate submissions increase from an average of 5158 articles per month in 2012 to 7931 articles per month in 2013 and 7057 in 2014 [36]. Although NIH operates in a much different context than the MS Society of Canada, the NIH experience highlights that the requirement itself is not a sufficient motivation to overcome perceived barriers to compliance. Policy enforcement is a necessary component in this process. Whereas the MS Society of Canada has implemented an OA policy, the mechanisms for enforcement are not explicitly outlined in that policy.

Whether mandated OA will move MS research to a more open dissemination environment remains to be seen. The movement to OA requires incentives for involvement [37]. In the case of researchers, for whom promotion and tenure decisions may be significantly impacted by the number of publications and the venue in which they are published, journal selection may be a critical choice. Authors select journals to publish in based on journal reputation, impact factor, and turnaround time to publication [22,28,38]. Watson [39] found that the authors are confused by the notion of OA, reluctant to participate, and confounded by the myriad of choices they are presented with when trying to publish OA.

Surveys have shown that researchers have generally favorable views of OA and its benefits for both the public and the scientific community [40,41]. However, there are challenges to making work available through both green and gold roads to OA. Initial challenges include informing researchers about the concept of OA itself, as many scientists and researchers have no direct mechanisms in place to become more informed about these issues and may not recognize the necessity of doing so [42].

### Study Limitations

As this study relied on bibliographic analysis rather than contacting researchers directly to provide a list of articles, the 758 articles used in the analysis may not have been directly produced as a result of the MS Society of Canada funded research. Any participating authorship by the 77 researchers during the time of funding by the Society was included in the study as long as the article was related to the topic of MS.

### Conclusions

The prevalence of OA literature produced by the MS Society of Canada researchers has remained consistently low between 2009 and 2014. Of the 758 articles, 288 are available OA. Most OA articles were made available without evidence of APC payment. Although APCs in hybrid journals was significantly higher than those associated with pure gold journals, the use of APCs for publication was low among this group.

The recent implementation of the MS Society of Canada’s OA policy may increase OA publication within this field. Future research should include prevalence of OA of newly funded research as a means of determining policy impact and effectiveness.

## Abbreviations

APC: article processing charge
CRKN: Canadian Knowledge Research Network
IR: Institutional Repository
MS: multiple sclerosis
NIH: National Institutes of Health
OA: open access
